# Constructing Compact Takagi-Sugeno Rule Systems: Identification of Complex Interactions in Epidemiological Data

**DOI:** 10.1371/journal.pone.0051468

**Published:** 2012-12-14

**Authors:** Shang-Ming Zhou, Ronan A. Lyons, Sinead Brophy, Mike B. Gravenor

**Affiliations:** Centre for Health Information Research and Evaluation, College of Medicine, Swansea University, Swansea, United Kingdom; Umeå University, Sweden

## Abstract

The Takagi-Sugeno (TS) fuzzy rule system is a widely used data mining technique, and is of particular use in the identification of non-linear interactions between variables. However the number of rules increases dramatically when applied to high dimensional data sets (the *curse of dimensionality*). Few robust methods are available to identify important rules while removing redundant ones, and this results in limited applicability in fields such as epidemiology or bioinformatics where the interaction of many variables must be considered. Here, we develop a new parsimonious TS rule system. We propose three statistics: *R*, *L*, and *ω*-values, to rank the importance of each TS rule, and a forward selection procedure to construct a final model. We use our method to predict how key components of childhood deprivation combine to influence educational achievement outcome. We show that a parsimonious TS model can be constructed, based on a small subset of rules, that provides an accurate description of the relationship between deprivation indices and educational outcomes. The selected rules shed light on the synergistic relationships between the variables, and reveal that the effect of targeting specific domains of deprivation is crucially dependent on the state of the other domains. Policy decisions need to incorporate these interactions, and deprivation indices should not be considered in isolation. The TS rule system provides a basis for such decision making, and has wide applicability for the identification of non-linear interactions in complex biomedical data.

## Introduction

In the use of health informatics, one way to support public services planners in making decisions under uncertainty is to provide decision models that are robust and have excellent predictive performance. Preferred models tend to be as simple as possible while providing a good fit to the system’s behaviour (Occam’s razor [1]). The benefits of more parsimonious models lie in that they 1) are easier to interpret; 2) are more likely to avoid over-fitting; 3) can be better generalised; and 4) use fewer computing resources.

Fuzzy logic has become one of the cornerstones for characterising uncertainty in system modelling and data mining [2]–[5]. The TS fuzzy rule model [5] is commonly used and has two main advantages. The first is its representative power, being able to describe a highly nonlinear system with simple *local linear models* (LLMs). The second is its connections with linear-in-parameters models, so that linear system modelling techniques can be applied. In constructing a TS fuzzy model, the input space is decomposed into fuzzy regions, and LLMs are used to approximate the system in each individual region. The overall system output is obtained by fusing these subsystems. In this manner, an interaction between variables, whereby the effect on an output measure of a given level of a variable is dependent on the level of one or more other covariates, is easily revealed. The interaction will be represented by notably different output rules at different combinations of variable levels (regions of the data space).

Unfortunately, the bottleneck of using TS fuzzy systems in many practical applications is the high dimension of information space, which necessitates a large number of LLMs (the *curse of dimensionality*) [6]–[11]. As a result, the use of TS models in data rich fields such as epidemiology, medical statistics, bioinformatics and health informatics is limited. This is an unfortunate drawback. In such fields, a complex interaction between variables is expected. Few epidemiological indices can be treated in isolation, and a statistical method of analysis must consider how the effect of different levels of one risk factor can be dependent on or modified by the level of many other factors. This is precisely the strength of the ‘if-then’ TS rule system. High order interactions can easily be specified, without the need of a complex overall model structure (involving non linear functions for example). A method for constructing a compact, but robust, rule base for the TS model would therefore be of practical use.

In the wide field of fuzzy modelling, there are several methods proposed to tackle the *curse of dimensionality*, for example, *hierarchical fuzzy systems* [12]–[14]. However, the hierarchical decomposition is not suitable for TS fuzzy models in studies such as ours. There are several reasons for this. Although the hierarchical method applied to TS fuzzy systems can decrease the exponential growth of fuzzy rules, the exponential growth of parameters remains inherent [14]. Hence studies based on hierarchical fuzzy models have to face the difficulty of interpreting relatively more complexity in the rules themselves [15]. Specifically, building such a hierarchical TS fuzzy system corresponds to moving the complexity of the system from the antecedent (i.e., the need of *m^n^* rules) to the consequent part, as a result, each rule is more complicated in hierarchical system than in the corresponding standard TS (with LLMs) system [12]. Also importantly, there is great difficulty in handling the intermediate variables introduced by the hierarchical structures [14]. The intermediate variables usually do not possess any physical meaning and may go outside their definition domain, consequently causing a loss of linguistic interpretability [10] [14] [16]. With hierarchical fuzzy systems, it can be impossible to gain interpretations of relationships between input variables and outcome for practical applications. Lastly, hierarchical decomposition of problem is not always trivial, and in many applications cannot be accomplished [12].

**Figure 1:**
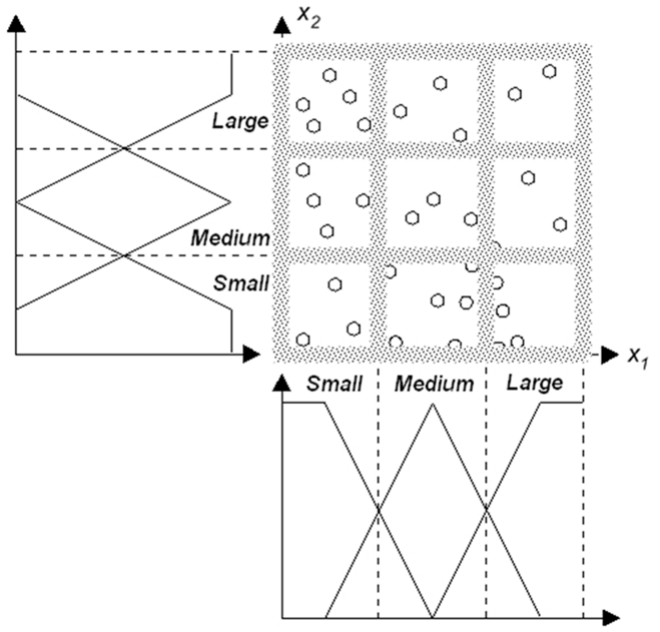
The input space partitioning for TS fuzzy model. Each region representing combinations of the variables *x_1_* and *x_2_* is described by an “*if-then*” rule with local linear model.

In most biological or medical applications the aim of the modelling is not simply to forecast, but to gain an understanding of precisely how certain variables interact, and to identify the key variables and levels of variables. This ability is offered by the standard TS approach due to the simple LLMs that are applied in each section of data space. We therefore seek a solution to the dimensionality problem for the TS model, rather than the use of hierarchical systems, for a very important reason: we wish to preserve the TS model transparency and ease of interpretation, a particular strength when trying to interpret complex biological and medical data. However the problem of the large rule base remains the key issue to be overcome if these systems are to be applied to high dimensional problems [6]–[10].

**Figure 2:**
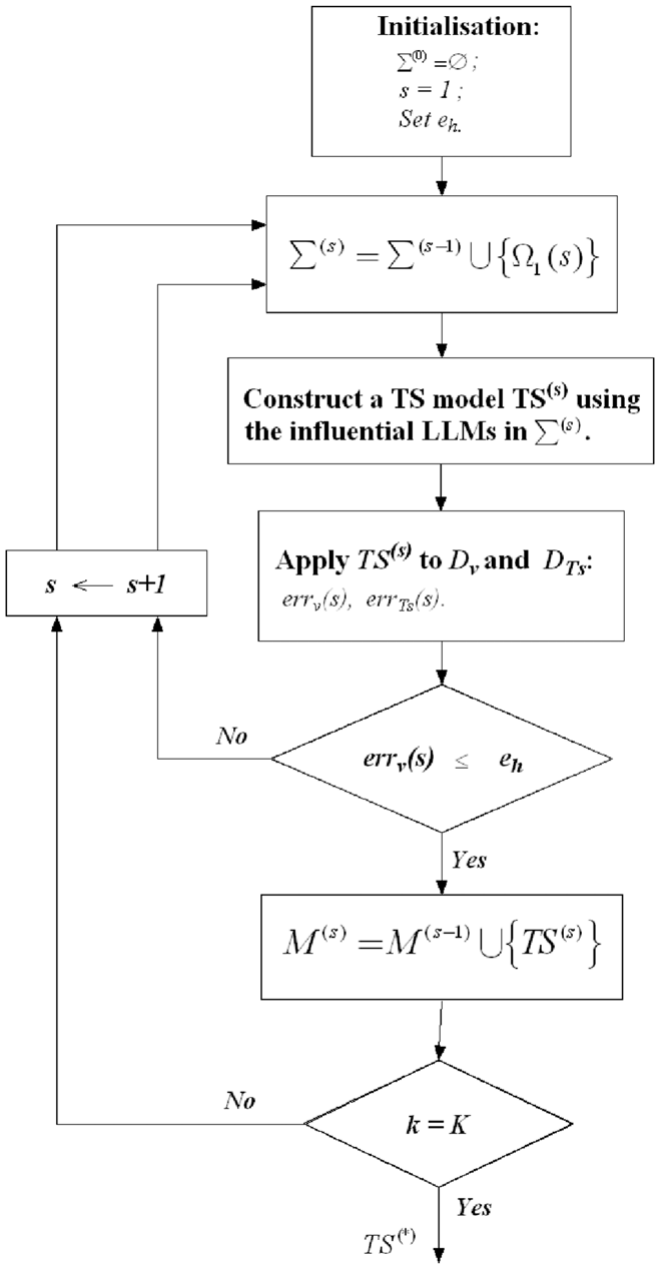
Forward selection procedure for selecting important LLMs.

Currently, the *SVD-QR* with column pivoting algorithm has been proposed to perform rule selection for a parsimonious fuzzy rule-base [17]–[19]. Unfortunately, some existing studies have shown great sensitivity to the chosen effective matrix rank (MR) values, so that different estimates of the MR often produce dramatically different rule-reduction results [19]. Here, we attempt to derive a general method for identifying the parsimonious set of TS fuzzy rules. We apply the method to a data set describing the complex relationship between a range of measures of childhood deprivation, and educational achievement. This is a typical data set available to policy planners and epidemiologists, where some strong general trends are expected alongside very complex and subtle interactions between risk factors.

**Figure 3:**
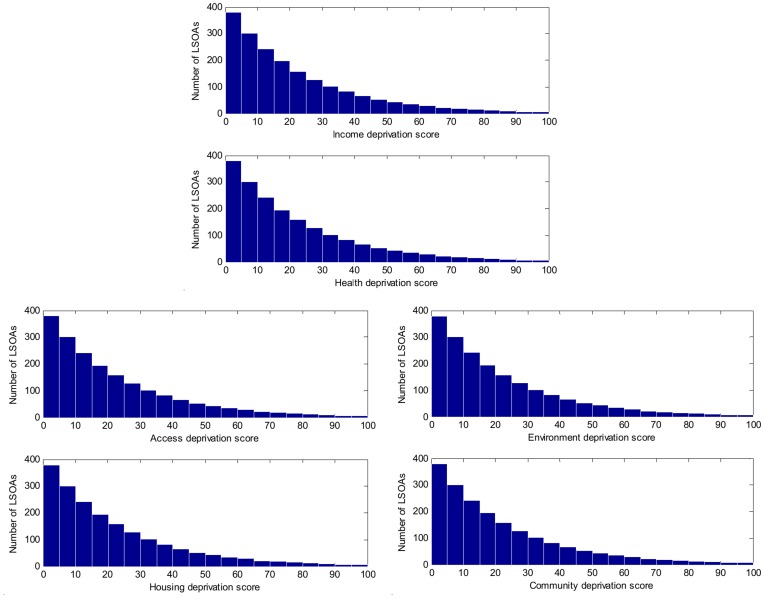
Distributions of observed scores on individual deprivation domains across all small geographic areas (LSOAs).

It is widely accepted that deprivation is a key component of social inequality, for example, rates of admission to hospital for cardiovascular conditions are influenced by socioeconomic deprivation [20], and the relationship between deprivation and educational achievement in childhood is crucial to understanding the substantial impact of deprivation on later outcomes in adulthood [21]. The “*Independent Inquiry into Inequalities in Health Report*” [22] in the UK stimulated studies of the complex relationship between poverty and health, and the *Welsh Index of Multiple Deprivation* (WIMD) was designed as part of the Neighbourhood Statistics programme in England and Wales [23]. This index, like other complex measures of deprivation, is based on the assumption that an overall measure of deprivation is a combination of different domains. For example, substandard housing or low income may contribute to poor health, but poor health is also a deprivation factor on its own right. WIMD is calculated for small geographical units called *Lower Layer Super Output Areas* (LSOA, with around 1500 people), which were generated by the Office for National Statistics by taking into account population size, mutual proximity, and social homogeneity and are designed to be permanent. WIMD is an important resource for the distribution of monies for public services and there has long been interest in whether such area-level indices of socioeconomic position are actually useful for predicting health outcomes or educational attainments in many countries [24]–[27]. Existing studies tend to focus on individual domains, and have rarely used high dimensional indices of multiple deprivations to explore the inherent interactions. Here we use the TS model system and new rule selection criteria to explore whether multiple indices of deprivation can influence child educational outcomes and how these multiple indices interact with each other to influence educational outcome for different categories of children.

**Figure 4:**
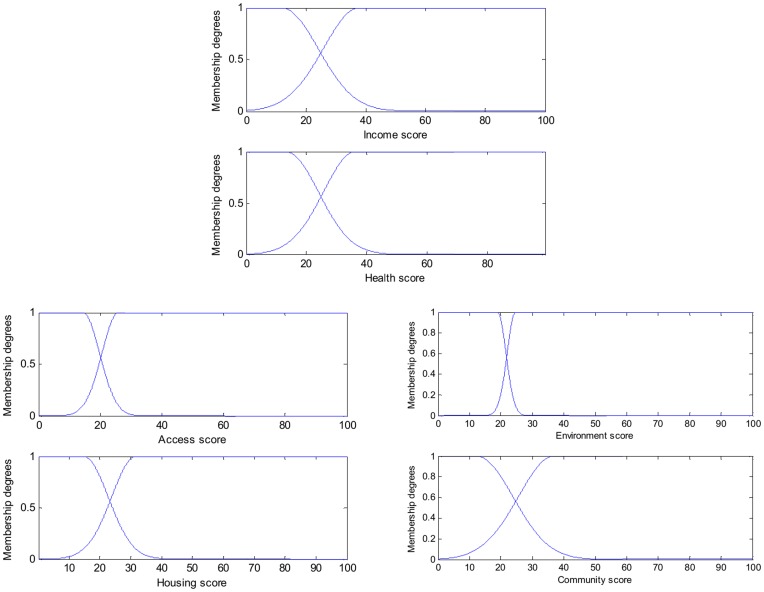
Fuzzy sets characterising the membership – low deprivation or highly deprivation within each child deprivation domain.

**Table 1:** Cut-off points for low-deprivations and high-deprivations on input domains.

Input variables	Low Deprivation core score range	Deprived area core score range
Income	[0, 11.91]	[37.95, 100]
Health	[0, 13.22]	[36.29, 100]
Access	[0, 14.41]	[25.97, 100]
Housing	[0, 14.74]	[31.69, 100]
Environment	[0, 18.97]	[24.66, 100]
Community	[0, 12.39]	[37.00, 100]

## Materials and Methods

### 2.1 TS Fuzzy Modelling Framework and Current Rule Selection Method

The TS fuzzy model decomposes the input space into fuzzy regions, approximates the system in every region by a LLM, and then combines these LLMs into an overall system output (Figure 1). A TS fuzzy model is expressed as follows [5]:


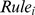
: If 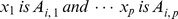 then
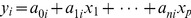
(1)


where, 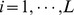, 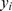 is the output variable of the *i*th rule, 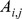 is a fuzzy set of the *j*th domain in the *i*th rule, and 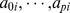 are consequent coefficients of the *i*th rule. Compared with a Mamdani fuzzy model, the rule consequent part is replaced by an affine linear function of input variables. As such, each rule can be considered as a *local linear submodel*. An overall output *y* is produced by fusing together these *LLMs* 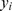 as
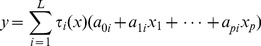
(2)where



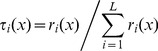
(3)is the normalized firing strength of the *i*th rule, and 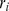 is usually defined by
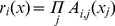
(4)


in which the 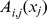 is the membership function of the fuzzy set 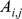. The overall system model (2) is also called the *global* model. The coefficients determine the size and direction of the effects in the local fuzzy region.

**Figure 5:**
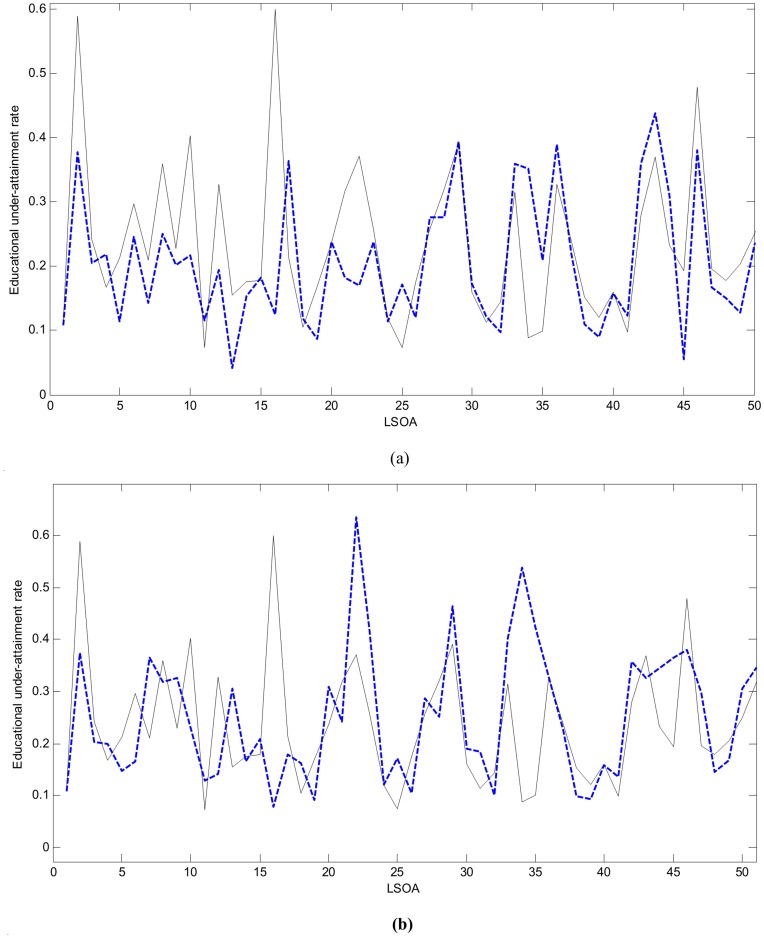
Prediction results on 50 LSOA areas (from the testing set, *D_Ts_*). (a) By the initial system model (64 LLMs); (b) By a model with 6 LLMs, selected using the *ω* -values in our proposed rule selection procedure. The solid line represents the observed education deprivation scores while the dashed line represents the model.

**Figure 6:**
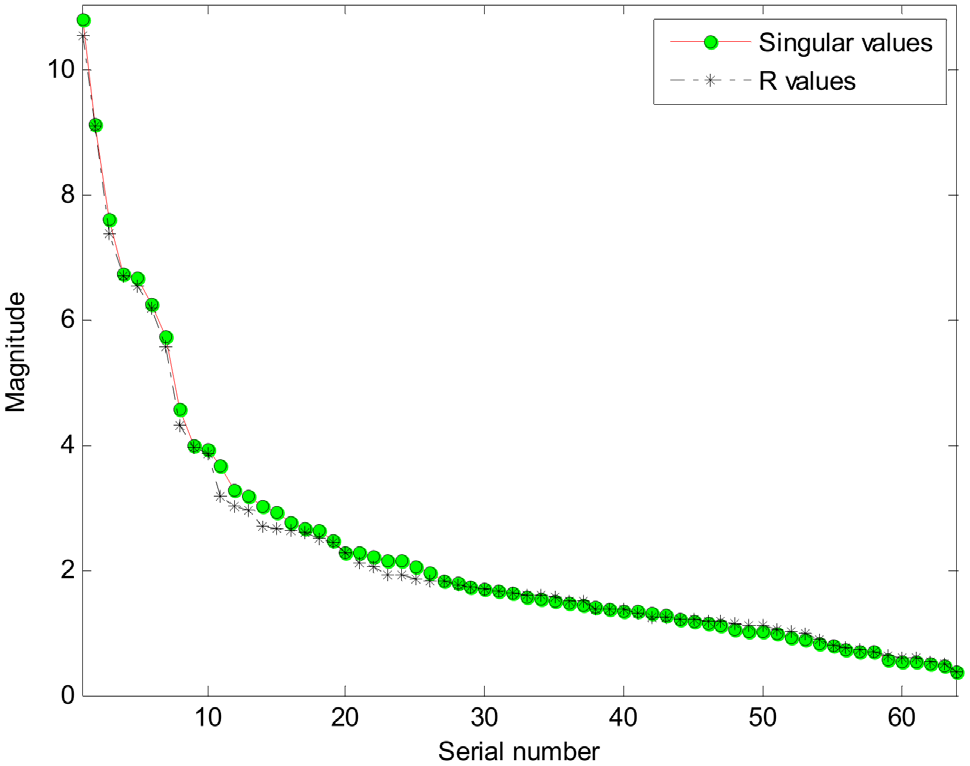
The *R*-values and singular values of TS fuzzy rules.

**Figure 7:**
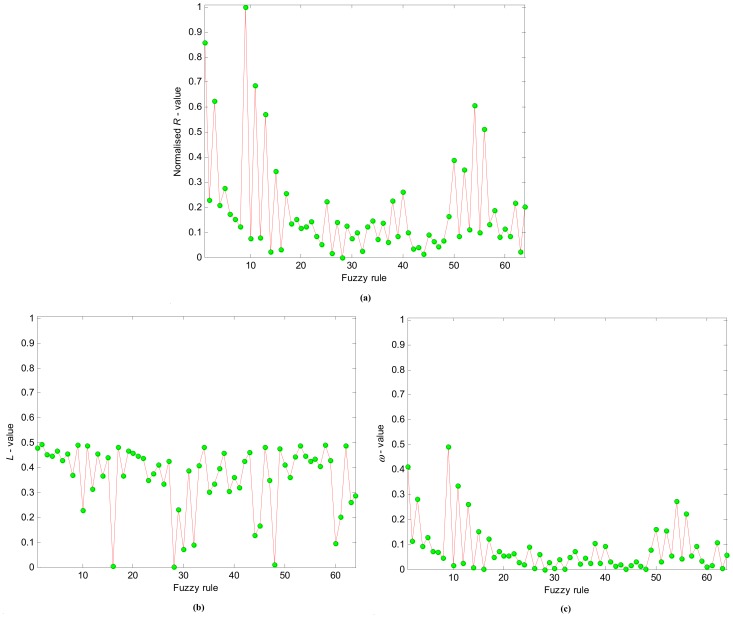
The *R*-values (a), *L*-values (b) and *ω*-values (c) of TS fuzzy rules in natural order.

**Table 2:** Rule ranking results in terms of R-values, L-values, and ω-values (Numeric values represent rule IDs).

Index	Rule ranking results
R-values	9 1 11 3 54 13 56 50 52 15 5 40 17 2 38 25 62 4 64 58 6 49 19 7 34 22 27 36 18 57 29 8 21 33 20 60 53 41 31 55 45 51 61 39 23 59 12 10 30 35 48 46 37 24 47 43 42 16 32 14 63 26 44 28
L-values	2 9 58 53 11 62 34 17 46 1 49 19 5 43 38 20 13 7 3 54 21 4 52 15 22 56 59 6 42 55 27 50 25 33 57 37 31 24 8 18 14 40 51 47 23 36 26 41 12 39 35 64 63 29 10 61 45 44 60 32 30 48 16 28
ω-values	9 1 11 3 54 13 56 50 52 15 5 17 2 62 38 40 4 58 25 49 6 19 34 7 22 27 64 53 21 20 57 33 18 36 8 55 31 59 41 51 46 29 23 39 12 37 35 24 43 10 61 45 47 42 60 14 26 63 30 32 44 48 16 28

Given *N* input-output data pairs 
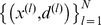
, the matrix
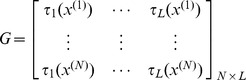
(5)is the *firing strength matrix*, in which each column corresponds to one fuzzy rule. Promisingly, the equation (2) can also be viewed as a *linear-in-parameters* regression model,



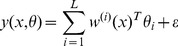
(6)where 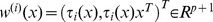 and 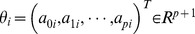, 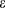 is the random noise. Furthermore, the (2) can be expressed in matrix form as follows,
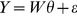
(7)


where 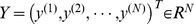, 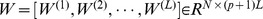, 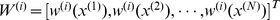 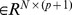 is called the *weight matrix*, 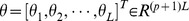. It can be seen that if the *basis functions* (BFs) 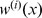 are fixed, then (6) or (7) becomes linear with respect to parameters 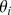.

**Table 3:** LLM selection results by FS procedure in terms of R-values, L-values, and ω-values (Numeric values in the 2nd column represent rule IDs).

Index	Influential LLMs selected	Number of LLMs	RMSE_v_	RMSE_t_
R-values	9 1 11 3 54 13 56 50 52 15 5 40 17 2 38 25 62 4	18	0.1101	0.1138
L-values	2 9 58 53 11 62 34 17 46 1 49 19 5 43 38 20 13 7 3 54 21 4 52 15	24	0.10	0.1176
ω-values	9 1 11 3 54 13 56 50 52 15 5 17 2 62 38	15	0.1104	0.1114

Each column of the firing strength matrix *G* corresponds to one fuzzy rule. Important rules correspond to the columns of the matrix that are linearly independent of each other [19]. The SVD (*singular value decomposition*) of *G* plays an important role in rule selection. The redundant fuzzy partitions (corresponding to the linearly dependent or zero-valued columns) are associated with near zero singular values of *G*. The smaller the singular values, the less influential the associated fuzzy rules. The *SVD-QR* with column pivoting algorithm has been popularised in identifying the most important fuzzy rules from a given rule base.

**Table 4:** Rule ranking results by *SVD-QR* with column pivoting algorithm.

Matrix rank	Rule ranking
4	38 46 49 42 5 6 7 8 9 10 11 12 13 14 15 16 17 18 19 20 21 22 23 24 25 26 27 28 29 30 31 32 33 34 35 36 37 1 39 40 41 4 43 44 45 2 47 48 3 50 51 52 53 54 55 56 57 58 59 60 61 62 63 64
5	45 46 38 49 42 6 7 8 9 10 11 12 13 14 15 16 17 18 19 20 21 22 23 24 25 26 27 28 29 30 31 32 33 34 35 36 37 3 39 40 41 5 43 44 1 2 47 48 4 50 51 52 53 54 55 56 57 58 59 60 61 62 63 64
6	45 62 46 38 49 42 7 8 9 10 11 12 13 14 15 16 17 18 19 20 21 22 23 24 25 26 27 28 29 30 31 32 33 34 35 36 37 4 39 40 41 6 43 44 1 3 47 48 5 50 51 52 53 54 55 56 57 58 59 60 61 2 63 64

**Table 5:** LLM selection results by *SVD-QR* with column pivoting algorithm.

Rank	Influential LLMs selected	Number of LLMs	RMSE_v_	RMSE_t_
4	38 46 49 42 5 6 7 8 9 10 11 12 13 14 15 16 17 18 19 20 21 22 23 24 25 26 27 28 29 30 31 32 33 34 35 36 37 1 39 40 41 4 43 44 45 2 47 48 3 50 51 52 53 54 55 56 57 58 59 60 61 62 63	63	0.10877	0.1046
5	45 46 38 49 42 6 7 8 9 10 11 12 13 14 15 16 17 18 19 20 21 22 23 24 25 26 27 28 29 30 31 32 33 34 35 36 37 3 39 40 41 5 43 44 1 2 47 48 4 50 51 52 53 54 55 56 57 58 59 60 61 62 63	63	0.10877	0.1046
6	45 62 46 38 49 42 7 8 9 10 11 12 13 14 15 16 17 18 19 20 21 22 23 24 25 26 27 28 29 30 31 32 33 34 35 36 37 4 39 40 41 6 43 44 1 3 47 48 5 50 51 52 53 54 55 56 57 58 59 60 61 2 63	63	0.10877	0.1046

In short, the algorithm works as follows. First, calculate the SVD of firing strength matrix *G* in 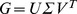 where 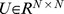, 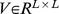, and estimate its effective rank from 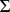. Next calculate a permutation matrix 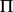 such that the columns of the matrix *G_r_* in 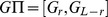 are independent. The actual rule selection is the calculation of the permutation matrix that extracts an independent subset of columns *G_r_*, assuming to correspond to the most important rules. This algorithm was originally proposed by Golub et al for subset selection in regression analysis [28], and has been used to select hidden nodes in a feed forward neural network [29]. However, in practical applications, one needs to choose a necessary effective rank for this algorithm. The negative consequence is that different choices of the rank often produce dramatically different rule reduction results [19] [30].

**Figure 8:**
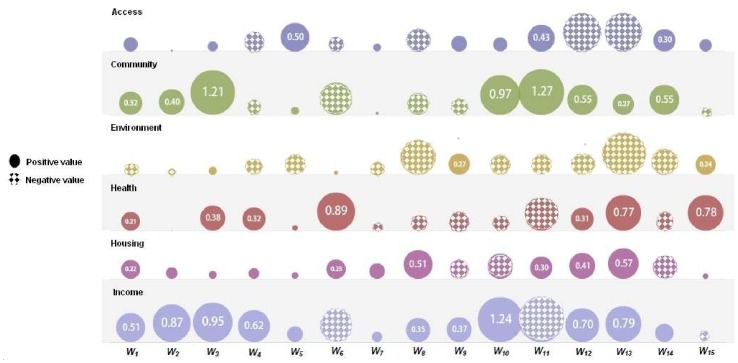
The coefficients of the 15 local linear models in the constructed TS system model.

**Table 6:** Examples of LSOAs dominated by each fuzzy rule.

Encrypted LSOA code	Income score	Health score	Accessscore	Housing score	Environmentscore	Communityscore	Which fuzzy rule dominates	Actual EUR	Predicted EUR
W0094	9.7	1.5	60.8	7.4	5.3	2.9	W_1_	0.07	0.18
W0736	1.4	4.5	15.5	6.7	0.5	0.8	W_2_	0.10	0.10
W0206	4.4	4.4	44.3	9.4	54.5	5.8	W_3_	0.17	0.15
W1627	0.5	2.0	14.0	1.0	49.1	5.2	W_4_	0.07	0.08
W1739	88.6	62.7	7.8	71.2	14.1	95.0	W_5_	0.41	0.42
W0689	11.2	6.5	66.2	29.2	8.7	3.4	W_6_	0.21	0.18
W1704	77.9	41.7	1.0	59.6	36.4	44.1	W_7_	0.32	0.37
W0941	42.4	48.1	8.7	8.1	3.2	36.6	W_8_	0.25	0.34
W0886	63.2	71.2	4.2	9.5	86.4	87.5	W_9_	0.43	0.42
W0475	5.3	0.4	83.5	31.5	50.6	1.0	W_10_	0.19	0.15
W1264	5.5	13.0	4.4	48.4	9.2	8.7	W_11_	0.33	0.23
W0798	11.7	32.8	9.8	16.8	2.4	13.0	W_12_	0.28	0.22
W0415	3.3	6.7	2.9	6.2	10.6	30.8	W_13_	0.15	0.10
W0862	100.0	96.6	22.7	44.1	2.7	95.8	W_14_	0.50	0.54
W1711	37.6	5.3	1.6	44.0	20.4	36.2	W_15_	0.30	0.27

**Table 7:** Fuzzy sets (high or low deprivation) associated with each local linear model in the TS system model constructed by *ω*-value index (D = high deprivation score, ‘ – ‘ = low deprivation score).

New rule ID	Income score	Health score	Access score	Housing score	Environment score	Community score
W_1_	–	–	D	–	–	–
W_2_	–	–	–	–	–	–
W_3_	–	–	D	–	D	–
W_4_	–	–	–	–	D	–
W_5_	D	D	–	D	–	D
W_6_	–	–	D	D	–	–
W_7_	D	D	–	D	D	D
W_8_	D	D	–	–	–	D
W_9_	D	D	–	–	D	D
W_10_	–	–	D	D	D	–
W_11_	–	–	–	D	–	–
W_12_	–	D	–	–	–	–
W_13_	–	–	–	–	–	D
W_14_	D	D	D	D	–	D
W_15_	D	–	–	D	–	D

**Table 8:** Coefficients of each local linear model in the TS system model selected by *ω*-value index*.

New rule ID	Constant term	Income score	Health score	Access score	Housing score	Environment score	Community score
W_1_	2.64	**0.51**	0.21	0.13	0.22	−0.08	0.33
W_2_	8.27	**0.87**	0.00	0.00	0.08	−0.02	0.40
W_3_	4.00	0.96	0.39	0.06	0.04	0.04	**1.22**
W_4_	18.67	**0.62**	0.33	−0.20	0.07	−0.16	−0.10
W_5_	20.75	0.16	0.02	**0.50**	0.03	−0.25	0.04
W_6_	17.17	−0.61	**0.89**	−0.12	0.23	−0.01	−0.64
W_7_	25.46	0.07	−0.01	0.04	**0.15**	−0.09	−0.01
W_8_	36.03	0.36	−0.10	−0.32	0.51	−**0.76**	−0.29
W_9_	22.43	**0.38**	−0.17	0.15	−0.23	0.28	−0.16
W_10_	23.09	**1.24**	−0.13	0.12	−0.39	−0.25	0.97
W_11_	12.28	−1.27	−0.68	0.44	0.31	−0.23	**1.28**
W_12_	6.71	0.70	0.32	−**0.83**	0.42	−0.28	0.56
W_13_	0.65	0.80	0.77	−0.76	0.57	−**1.10**	0.27
W_14_	4.53	0.20	−0.16	0.31	−0.33	−0.42	**0.55**
W_15_	15.94	−0.06	**0.78**	0.09	0.02	0.25	−0.03
**Mean**	14.58	**0.33**	0.16	−0.03	0.11	−0.20	0.29
**95%CI**	(8.98, 20.18)	(−0.03, 0.68)	(−0.07, 0.4)	(−0.23, 0.18)	(−0.04, 0.27)	(−0.40, −0.01)	(−0.01, 0.60)

* Highest absolute coefficient values are highlighted of each rule. Note that a positive coefficient represents a positive association between the level of deprivation and the education under-achievement rate (i.e. a positive association between measures of affluence and educational success).

### 2.2 Modification of the Current Method: The Index of R-values of TS Fuzzy Rules Considering the Effects of Rule Antecedent Parts

In order to avoid the estimation of the effective rank values, we apply the pivoted *QR* decomposition to the firing strength matrix *G*. The *QR decomposition* (also called *QR factorization*) of a matrix is a decomposition of a matrix *A* into a product *A* = *QR* with an orthogonal matrix *Q* and an upper triangular matrix *R*, which is often used to solve the linear least squares problem [31]. In this paper, the absolute values of the diagonal elements of matrix *R* in *QR* decomposition are called the *R*-values of *G*. The *R*-values tend to track the singular values of the matrix *G*, so these *R*-values can be used in rule ranking as follows:

#### Step 1

Calculate the *QR* decomposition of *G* and obtain the permutation matrix 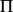 via 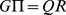, where *Q* is an orthogonal matrix, *R* is an upper triangular matrix. The absolute values of the diagonal elements of *R*, denoted as 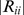, decrease as *i* increases and are named as *R*-values.

#### Step 2

Rank fuzzy rules in terms of the *R*-values and the permutation matrix 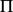. Each column of 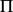 has one element taking the value 1 and all the other elements taking the value 0. Each column of 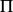 corresponds to a fuzzy rule. The numbering of the *j*th most important rule in the original rule base is the same as the numbering of the row where the “1” element of the *j*th column is located. For example, if the “1” of the 1^st^ column is in the 4^th^ row, then the 4^th^ rule is the most important one and its importance is measured as 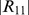. The rule corresponding to the first column is the most important, and in descending order the rule corresponding to the last column is the least important.

### 2.3 A New Index for TS Fuzzy Rules: *L-values* take into Account the Effects of Rule Consequent Parts

The *R*-values only take into account the rule base structure (focusing on the rule antecedent parts). An alternative approach is to consider the effects of rule consequents [30]. The so-called 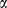-values of fuzzy rules [30] have been proposed to consider the contribution of rule consequent parts in constructing parsimonious linguistic type fuzzy models whose consequent parts are constants. These 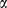-values of fuzzy rules are actually the absolute values of consequent constants. One may naturally infer that the indices 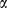 *-values of fuzzy rules* [30] can be extended to TS fuzzy models with LLM consequents, where the new index for TS model is defined as sum of absolute values of consequent parameters 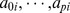 in (1) or length of the vector 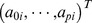. However, our experiments suggest this is not the case, with a tendency for system output to exceed domain range and hence poor generalization performance. If one considers the differences between the TS fuzzy model (1) and the linguistic type fuzzy model whose consequent parts are constants, it can be found that their submodels exhibit completely different interactions with the global models [7] [9] [32]. Instead, we propose a new index for ranking TS fuzzy rules by considering the contribution of the LLMs, termed as *L*-values for TS rules.

*Definition*. *L*-value of TS fuzzy rule 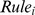 is
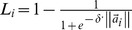
(8)


where 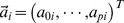, 
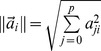
, 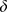 is a parameter defined by user to expand or shrink differences among *L*-values.

### 2.4 A Further New Index for TS Fuzzy Rules: *ω-values* take into Account the Effects of Both Rule Antecedent Parts (R) and Consequent Parts (L)

In order to consider both the TS rule base structure information and the contribution of LLMs for rule ranking, we propose a further index:
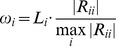
(9)


where 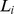 and 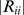 are the *L*-value and *R*-value of *Rule_i_* respectively.

### 2.5 Local Linear Model Selection and Implementation

The standard system modelling technique usually involves tasks of model construction and evaluation of the generalization performance. The datasets used for the two tasks should not be the same. In our study, one additional task, *model selection*, is involved. So the data is split into three subsets. 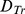 is used to identify the system parameters in training a TS fuzzy model 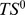. The performance of the trained 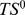 is measured in terms of the error index 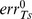 obtained by applying to the testing samples in 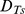. The data subset 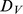 is used to validate the selected fuzzy rules for constructing a compact TS model. The 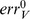 measures the validation performance by applying to the validation samples in the data set 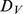.

The *R*-values, *L*-values, and 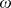-values can be used to identify the most influential TS fuzzy rules that ensure the smallest possible model explains the available data well. First, assume 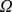 to be the results obtained from a rule ranking index:


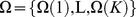
(10)

where *K* denotes the number of rules in the initial model 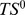. The rule importance denoted by 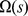 decreases as *s* = *1*, *2*, ···, *K* correspondingly. Let 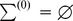 be the rule subset that includes recursively selected rules.

*The Forward Stepwise (FS) Procedure* is a heuristic that starts with an empty set of TS fuzzy rules (i.e. 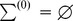):

1. Set, 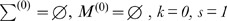, assign a model error tolerance threshold 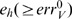;
2. Select the most important TS fuzzy rule(s);
3. Construct a model 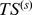 using the influential fuzzy rules;
4. Apply 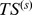 to the validation dataset *D_V_* and the test dataset *D_Ts_* to obtain new root-mean-square -errors (RMSEs): 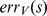 and 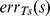;
5. If 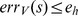, then put the model 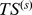 into 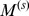 and increase s by 1 and go to Step 2. If 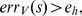 increase s by 1 and go to Step 2;
6. If *s = K*, then stop the process and select a 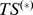 in 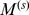 with the most compact rule-base as the final model and use the corresponding 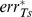 as the measure of generalization performance for 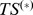.

Using the FS procedure, one at a time, the highest ranked LLM is added to 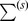. The models with 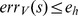 are added to the model-base 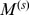, and the procedure continues until every rule has been assessed. Then the most compact model 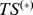 among 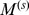 is selected as the final global model. That is to say, the model 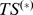 has the smallest number of LLMs used, at the same time the 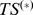 achieves satisfactory approximation ability with 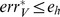 (see Figure 2). We note that because *e_h_* 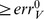, the termination of the FS procedure is guaranteed.

Our implementation of the methodology is as follows (we note a similar backward selection procedure can be defined):

**Step 1**. Initialise the input space partition.

**Step 2.**Train a TS model by a system modelling technique.

**Step 3**. Rank the rules of the trained TS model in terms of the new indices.

**Step 4.** Select most influential LLMs using the FS procedure.

**Step 5**. Select the final compact model indicated via the FS procedure.

A linear least squares method is used in this study to identify the parameters in (7) for *Step 2*. For TS models with hyper-parameters, other methods are available (e.g. ANFIS [33]). We note that there is a choice to be made regarding the error threshold *e_h_* in the above scheme. *e_h_* controls the trade-off between global model accuracy and parsimony of the rule base. With specific data, a trial-and-error procedure is appropriate to determine how much the global model accuracy can be degraded at the expense of a compact rule base.

### 2.6 Data sets: WIMD Child Index Data and Linkage with Educational Outcomes

The *Child Index of Deprivation* is as a sub-index of the WIMD multiple deprivation index. The latest (2008) version comprises seven separate domains of deprivation relevant to children: *income*, *health*, *access to services*, *housing*, *physical environment*, *community safety* and *education* (*including skills and training*) [23]. Each domain score was developed in terms of a combination of relevant indicators. Selection of the indicators for each domain is based on up-to-date, comprehensive and robust criteria, and is available for the entire country at the LSOA small geographic area level. The significance of research on area-based effects lies in emphasising the need to focus public health and educational initiatives on the broader characteristics of places where disadvantaged people live, rather than simply on the people who live in these areas themselves [24].

The 2008 WIMD domains are held in the SAIL databank [34]–[35], a national electronic health research infrastructure. Each domain is scored on a level 0–100 (with 100 the highest level of deprivation), and is itself constructed from several indicators [23]. Since our outcome variable is educational achievement (see below) we omit the education deprivation index domain from the analysis (this index already contains summary information on education achievement). Of the remaining 6 domains, *Income* measures the extent of deprivation relating to income, at the small area level. It focuses on the proportion of children living in households with income below a defined threshold or claiming benefits relating to low incomes. *Health* captures the degree to which children are deprived of good health, as determined by the area prevalence of limiting long-term illness and low birth weight. *Housing* captures deprivation though a lack of central heating and overcrowding. *Physical Environment* measures environmental factors that may impact on quality of life, including air quality, emissions, flood risk and proximity to waste disposal and industrial sites. *Access to Services* measures deprivation resulting from a household’s inability to access a range of services, considered necessary for day-to-day living (average travel time to schools, libraries, leisure centres). *Community Safety* combines police recorded crime, numbers of youth and adult offenders and incidents of fire.

The SAIL databank currently holds individual record level data for pupils in all maintained schools in Wales between 2003 and 2008. In the UK, state education consists of 5 Key Stages (KS), and the National Curriculum sets out targets to be achieved in various subject areas at each stage. In this study, we focus on the child educational attainment at KS1 and KS2. The KS1 covers two years of schooling in maintained schools in England and Wales normally when pupils are aged between 5 and 7. The KS1 attainments are assessed in three subjects: mathematics, science and either English or Welsh. If a pupil attains a satisfactory score for all the three subjects, this pupil is considered to have reached the expected KS1. The KS2 covers four years of schooling in maintained schools in England and Wales when pupils are aged between 7 and 11. The KS2 attainments are assessed in a similar way as the KS1 attainments.

We define the overall *education under-attainment rate*, at the LSOA level, as the total number of children achieving lower than expected levels (KS1 and KS2) divided by the total number of assessments made over the three year period 2005 to 2007. Then, using the code of each LSOA as system linkage field in the SAIL databank, the under-attainment rate is linked to the 2008 WIMD Child Indices, to explore how local components of deprivation interact to determine local area educational achievement.

## Results

First the 1896 LSOA samples were split into a training dataset 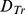 with 1400 samples, a testing dataset 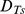 with 296 samples and a validation dataset 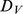 with 200 samples. The 1400 training samples were used to construct a TS model with 6 inputs representing the various indices of deprivation: *income* (*x_1_*), *health* (*x_2_*), *access to services* (*x_3_*), *housing* (*x_4_*), *physical environment* (*x_5_*), *community safety* (*x_6_*) and one output *education educational under-attainment rate* (*y*). The scales of independent variables on their domains are [0, 100], with 0 the least deprived and 100 the most deprived. The distributions of deprivation scores on individual domains across all the LSOAs are shown in Figure 3.

The fuzzy c-means unsupervised clustering algorithm [36] was used to partition input space. We note that other methods such as fuzzy learning vector quantization [37] can be used. Once the prototypes are generated, the membership functions are obtained by projecting the multi-dimensional prototypes on the input variable space [38]. The crucial points for the fuzzy sets in our study are shown in Figure 4. The cut-off points for low-deprivation and high deprivation score are shown in Table 1. These represent high degrees of certainty of high/low deprivation group membership. However, uncertainty emerges for the areas whose deprivation scores lie between the cut-offs, and different degrees of high/low membership are subsequently taken into account by the weights of the fuzzy rules.

The initial TS fuzzy model is composed of *2^6^ = 64* LLMs. The trained system model accurately predicts the impact of child deprivation on education achievement (Figure 5a, generalisation performance *RMSE* = 0.101). Next, we applied the proposed rule selection and reduction methods. Figure 6 shows that the *R*-values of TS rules track the singular values very well, and we conclude they are appropriate for the ranking of the fuzzy rules. These *R*-values in the original rule order are illustrated in Figure 7a, and the rule ranking results shown in Table 2.

Given the threshold *e_h_* = 0.111, and applying the FS procedure as addressed above, we select the significant LLMs in terms of the *R*-values. The rule selection results are given in Table 3, in which the *RMSE_v_* represents the *RMSE* of the TS model applied to validation samples, whilst the *RMSE_t_* is the *RMSE* of the TS model applied to testing areas. A parsimonious model is constructed by 18 LLMs identified from the original 64. This newly constructed compact TS model predicts the impacts of child deprivation at testing LSOAs with *RMSE_t_* = 0.1138. Using the new *L*-values (shown in natural order in Figure 7b), a TS model with 24 LLMs is obtained with generalization performance *RMSE_t_* = 0.1176 (Table 3). By taking into account the contributions from both rule premise parts and consequent parts, the new *ω*-values of TS fuzzy rules were obtained as shown in Figure 7c. As indicated in Table 3, 15 important LLMs were identified (generalization performance *RMSE_t_* = 0.1114). Figure 5b shows the prediction results of this compact model with only 15 LLMs, again showing good predictive power in modelling educational achievement.

Finally, as a comparison, we used the standard *SVD-QR* with column pivoting algorithm to select the important LLMs from the TS rule-base. Table 4 illustrates the rule ranking results under different assumed *SVD-QR* parameters. It can be seen that this approach is highly sensitive to the assumed parameter of matrix rank. As demonstrated in Table 5, under the FS procedure, the *SVD-QR* pivoted algorithm with matrix ranks 4, 5 and 6 all selects a remarkable 63 LLMs (testing sample *RMSE_t_* = 0.1046). The 3 TS fuzzy models constructed by the *SVD-QR* pivoted algorithm consist of the same LLMs in different orders, as a result the 3 TS fuzzy models are identical. Hence, our proposed indices provide a far more efficient means of identifying a parsimonious model, and the important LLMs.

## Discussion

Making predictions under uncertainty has become a critical activity in healthcare and planning of public services [39]. The TS fuzzy modelling scheme, based on a group of easily understandable *if-then* rules, is an ideal platform for modelling epidemiological outcomes. However, the method tends to use an oversized rule base to characterise the relationship between input variables and the dependent outcome. This can lead to statistical problems and is also cumbersome for decision making. Here, we have developed and tested new indices for ranking the rule-base in order to construct a compact model for predicting outcomes from many dimensional data, specifically how complex indices of child deprivation can be used to predict educational achievement. Our indices led to a model with 15 influential LLM rules, compared to 63 rules (out of a maximum of 64) obtained by the standard method. Hence there are very many redundant LLMs in the standard TS fuzzy rule-base, with a corresponding risk of over fitting and forecasting bias.

Because they are linear, the interpretation of the LLM in each TS rule is straightforward, being no different from a simple regression. The use of ‘*if-then*’ rules, even with a large rule base, is much more transparent from a decision maker’s perspective than, for example, multiple regression, with large numbers of interaction or non-linear terms, that are commonly used in the field. However, if there are a large number of (influential) rules, the overall model can of course become cumbersome, even this will simply reflect the number of interactions highlighted by the data and therefore a complex interpretation cannot be avoided.

We suggest that in order for TS model to identify the complex interactions of variables across local data regions, one needs to maintain the LLMs of a TS model that are able to represent the system behaviours in their corresponding subareas. In other words, these LLMs should fit the global model well in their local data regions, and result in fuzzy rule consequents that are local linearizations of a nonlinear system. The key to achieve this goal is to generate distinguishable membership functions for fuzzy sets in rule antecedents in which there is no much overlapping of neighbouring membership functions in the core area of each fuzzy set [7] [9] [38]. As shown in Figure 4, the fuzzy sets generated in this study can fufill this task.

It is widely recognised that children who have poorer childhood health and socioeconomic conditions tend to have lower educational attainments and other long term detrimental outcomes [40]–[44]. But there is less evidence on how this relationship changes across different health and socio-economic backgrounds. This issue becomes important because understanding the different effects of health and socio-economic factors on educational outcomes across different family backgrounds can lay a solid basis for developing different health, education socio-economic intervention programmes that target different groups of residents. We now discuss the interpretation of the child deprivation/education model in some details.

As discussed above, all fuzzy rules play a role in making prediction on all LSOAs, but with different weights (some effectively zero). But due to the lack of overlap of neighbouring membership in the core area of each fuzzy set (see Figure 4), we can take note of the dominating fuzzy rule in each case. Table 6 shows examples of LSOAs that are well described (“dominated”) by a single fuzzy rule (identified in terms of the *ω*-value index). The actual EUR is calculated as the total number of children achieving lower than expected levels (KS1 and KS2) divided by the total number of assessments made over the three year period 2005 to 2007 in this LSOA while the predicted EUR is obtained by using the TS model with the 15 LLMs to predict the educational performance for this LSOA. Table 7 illustrates the corresponding fuzzy sets associated with each LLM in the fuzzy region of data space (re-coded *W_1–15_*). Figure 8 illustrates the contribution of each domain (in terms of the size of the coefficients) for these 15 LLMs, and Table 8 gives a further summary, in which a positive coefficient represents a positive association between the level of deprivation and the education *under-achievement rate* (which is of course equivalent to a *positive* association between measures of affluence and educational *success*). These 15 rules can be used to cluster different geographical areas with similar characteristics. For example, LSOA W0736 is characterised by low deprivation on all domains, and is dominated by rule *W_2_*, which has the form:
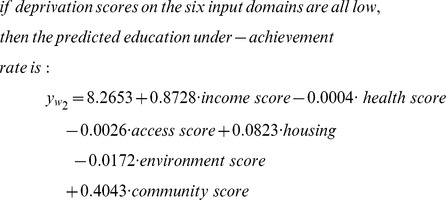



Thus, in an affluent area for which deprivation scores are all low, there is an overall low under-achievement rate, but with the following feature: The dominant factor (largest absolute coefficient value) influencing educational achievement is income, with a positive association with *income deprivation index*, and education under achievement.

Such an *income* effect is expected [40] [44]. But the rule base (Table 8) shows that it is not consistent across areas, often having a negligible effect. In one case there is an apparently strong negative effect. This is not easily explained, but may serve to draw attention to specific areas where additional domain specific knowledge needs to be applied. The rule *W_11_* is the dominant rule in areas characterised by highly deprived *housing* only, where it is also suggested by the model that improving *community safety* can make the most significant positive contribution to improvement of children educational outcomes. There are several other area categories, such as rules *W_6_*, *W_15_*, where *income* has a negligible effect, interestingly each of which is also characterised by a high *housing* deprivation score.

Currently very few studies have shown evidence of the effect of *community safety* on child educational outcomes. Here, we find several examples of strong *positive* associations with educational outcomes. Again however, the effect of *community safety* is not independent of the other characteristics of the area. For example, rule *W_6_* shows that for children experiencing highly deprived conditions in *access to services* and *housing* but good conditions on family *income*, *health*, *physical environment* and *community safety*, there is an apparent negative association between *community safety* and educational achievement. This may reflect a protective effect of supportive parenting, whereby concerns over *community safety* may be associated with general support at home.

The general observed effect of *health* is also expected [42] [45]. What is more interesting, again, is its influence in combination with other socio-economic factors for children from different backgrounds. For example, we find an interaction with the effects of *access* and *housing* deprivation. The strongest positive association between *health* and achievement is found in regions dominated by rule *W_6_*, characterised by high deprivation in *access* and *housing* only. In contrast, quite similar regions, dominated by rule *W_11_*, and thereby differing only in having low *access* deprivation, we find the least positive association between *health* and educational outcome.

Our study indicates that the *housing* deprivation index emerges as one of the strongest factors for positively influencing child educational outcomes in terms of average strength. But again, there is a very complex relationship when other details are taken into account. Rules *W_9_*, *W_10_*, *W_14_* suggest that “overcrowding” (a key feature of the housing deprivation score) may sometimes have a positive association with education, in the presence of several high deprivation scores. It is possible that children exposed to poorer *health* conditions (along with *income*, *physical environment* and *community safety*), but good *access to services* and *housing*, are more successful due to support provided by close family members.

*Access* and *environment* did not achieve strong *positive* associations under most circumstances. However, once the whole multiple dimensional data space is partitioned into fuzzy regions, some hidden relationships are revealed, such as a positive relationship between *access* deprivation and education achievement in some area types (rules *W_12_*, *W_13_*). Similarly rule *W_9_* yields the maximal *positive* association of *physical environment* with educational outcomes, while the rule *W_8_* presents the greatest *negative* association between the two variables, and yet such rules differ only in their typical level of *environmental* deprivation. The strength of the TS rule base is to highlight such apparent anomalies, while area-specific information would most likely be required by policy makers to resolve them.

Our study has demonstrated strong and complex relationships between measures of childhood deprivation and educational achievement, using a novel TS rule selection method. Consideration should be given to developing different policies on health and socio-economic intervention strategies for different categories of children to attempt to improve child education. For example, in the LSOAs dominated by the rules *W_1_ ∼ W_3_* public policy may need to focus on *income* (pockets of poverty in that area) and *community* development, while the areas fitting the rule *W_5_* perhaps should focus on *access* and *physical environment*. Our study provides an indication of factors which could help in guiding development of such policies and intervention strategies.

### Conclusions

Our study has demonstrated that the TS fuzzy model can capture complicated non-linear effects of interacting variables, whilst remaining (from a computational and, crucially, interpretation perspective) a relatively simple linear-in-variables approach.

This study has shown that novel combinations of the six of the domains in the WIMD (*income*, *health*, *geographical access to services*, *housing*, *physical environment*, *community safety*) produce excellent generalization performance in predicting child educational attainment at the small area level. These six forms of deprivation on individual domains interact synergistically to work as an effective predictor of the area based relationship between child deprivation and educational achievement. The relationship can be complex, and illustrates the advantage of the TS model approach. With the aid of the LLMs of TS system, we gained considerable insights into the patterns how the multiple health and socio-economic factors influence educational achievements for children from different backgrounds. Overall, the factor *income* exhibit strong *positive* associations with child educational outcomes for most of the children.

We suggest that there is very wide applicability of such a method, including the parsimonious rule selection scheme proposed in this paper, whenever the challenge is to combine the information from many domains into decision making tools and find relationships between such domains in complex observational data.
