# Correction: Constructing Compact Takagi-Sugeno Rule Systems: Identification of Complex Interactions in Epidemiological Data

**DOI:** 10.1371/annotation/3724e791-2df4-4c75-8d60-4327fe0eb6d0

**Published:** 2013-08-13

**Authors:** Shang-Ming Zhou, Ronan A. Lyons, Sinead Brophy, Mike B. Gravenor

Multiple funding organizations and grants were incorrectly omitted from the Funding Statement. 

The correct Funding Statement is: "This work was undertaken with the support of the Centre for the Development and Evaluation of Complex Interventions for Public Health Improvement (DECIPHer), a UKCRC Public Health Research Centre of Excellence. Funding from the British Heart Foundation, Cancer Research UK, Economic and Social Research Council (RES-590-28-0005), Medical Research Council, the Welsh Assembly Government and the Wellcome Trust (WT087640MA), under the auspices of the UK Clinical Research Collaboration, is gratefully acknowledged." 

